# Does evaluation of tumor volume or/both origination site better guide to optimal surgery for inverted papilloma?

**DOI:** 10.1016/j.bjorl.2019.10.005

**Published:** 2019-11-15

**Authors:** Muammer Melih Şahin, Metin Yılmaz, Mehmet Ekrem Zorlu, Mehmet Göcek, Mehmet Düzlü, Erolcan Sayar, Alper Ceylan

**Affiliations:** a Gazi University Faculty of Medicine, Department of Otorhinolaryngology/Head and Neck Surgery, Ankara, Turkey; b Gümüşhane State Hospital, Department of Otorhinolaryngology/Head and Neck Surgery, Gümüşhane, Turkey; c Gazi University Faculty of Medicine, Department of Pathology, Ankara, Turkey

**Keywords:** Inverted papilloma, Tumor origin site, Recurrence, Endoscopic approaches

## Abstract

**Introduction:**

Sinonasal inverted papilloma is noted for its high rate of recurrence. Staging systems aid to reduce recurrence and avoid excessive surgeries by guiding the selection of the optimal surgical approach.

**Objective:**

To evaluate the effectiveness of different endoscopic approaches in inverted papilloma by assessing tumor origin site and tumor volume.

**Methods:**

Krouse classification system that is based on tumor volume was used for staging; furthermore, tumor origin sites were grouped as lateral nasal wall, medial wall and other walls of maxillary sinus. The main treatment method for all patients was endoscopic sinus surgery. Endoscopic extended middle meatal antrostomy, endoscopic Caldwell-Luc and endoscopic medial maxillectomy were the additional surgery types performed in different combinations.

**Results:**

Fifty-five patients (42 male) with a mean 54.9 ± 14.4 years of age were included. 37 patients were diagnosed with advanced stage inverted papilloma (67.2 %). Recurrence was observed in 12 patients (21.8 %). In early stage lateral nasal wall origination, no recurrence was observed in the simple tumor resection group (0/10). In early stage medial wall origination, no recurrence was observed in the extended middle meatal antrostomy group (0/8). In advanced stage medial wall origination, the recurrence rates of extended middle meatal antrostomy, extended middle meatal antrostomy + endoscopic Caldwell- Luc and endoscopic medial maxillectomy were 100.0 %, 53.8 % and 13.6 %, respectively (*p* = 0.002). In advanced stage other walls of maxillary sinus origination, recurrence rates of extended middle meatal antrostomy + endoscopic Caldwell-Luc and endoscopic medial maxillectomy were 20 % and 16.6 %, respectively (*p* = 0.887).

**Conclusion:**

Tumor origin site, tumor stage and surgery types show an impact on recurrence. Despite the fact that tumor origin site singly could lead to appropriate selection of the surgery type in most cases, tumor stage carries substantial importance in selection of surgery type for sinonasal-inverted papilloma. An operation plan regarding both tumor volume and tumor origin site may aid surgeons in selecting optimal endoscopic surgical method to avoid recurrence or excessive surgeries.

## Introduction

Sinonasal inverted papilloma (IP) is a benign tumor and constitutes about 0.5–4% of the sinonasal region tumors. Inverted papilloma most commonly originates from the lateral nasal wall’s ectodermal Schneiderian mucosa. Rarely, it may also develop from the middle turbinate, middle meatus, paranasal sinuses and nasopharynx.^1^ IP is more common in men than in women. The incidence of IP is 2–6/1,000,000 and it is often seen in 5‒6th decades.^2^ Although the etiology is not elucidated well, factors such as allergy, smoking, chronic sinusitis and Human Papillomavirus (HPV) are incriminated.^3^ Despite being benign, it is an important nasal tumor diagnosis due to the risk of malignant transformation.^1^, ^3^ The first complaint of patients with IP is often nasal obstruction. Exophytic lesions involving one side which resembles nasal polyps are often observed in routine examinations. The exact diagnosis is made by histopathological examination.^4^ Several studies have reported that the recurrence rate of IP may range from 10 % to 25 %.^5^, ^6^ Complete surgical excision is the main desired treatment of IP.^7^ Although different surgical methods have been described, surgical approaches were evolved to pedicle-oriented endoscopic methods to avoid tumor recurrence in the recent years.^8^ Since it has been shown that the majority of the recurrent tumors reappeared from the tumor origin sites, different classifications that consider origin site have been defined.^7^, ^8^ Nevertheless, the most commonly used staging system is the Krouse classification system that based on tumor involvement sites (Table 1) and recent studies have shown that the tumor stage is closely related to recurrence.^9^ There are studies comparing the staging systems that are based on tumor volume in the literature.^10^ The aim of this study is to analyze the effectiveness of different endoscopic methods in sinonasal Inverted Papilloma (IP) by evaluating the impact of both tumor origin site and tumor volume.

**Table 1:** Krause staging system and distribution of the patients according to their stages.

	Krouse staging system for inverted papilloma^a^	Number	Percent
T1	The tumor is completely confined to the nasal cavity. It does not extend into sinuses or extranasal structures. Malignancy does not accompany.	0	0
T2	The tumor is confined to the medial wall of the maxillary sinus, ostiomeatal complex and ethmoid sinus. The nasal cavity may or may not be associated with involvement. Malignancy does not accompany.	18	32.7
T3	Disease involves the lateral, anterior, posterior or inferior walls of maxillary sinus or extension into frontal or sphenoid sinuses with or without involvement of medial portion of the maxillary sinus, the ethmoid sinuses or the nasal cavity. Malignancy does not accompany.	37	67.2
T4	This stage involves tumor spread outside the confines of nose and sinuses. This stage also includes malignancy.	0	0

a From Krouse.^17^

## Methods

In this study, patients who had undergone endoscopic sinus surgery for IP in our clinic between 2010–2017 were retrospectively analyzed. The ethics committee of clinical research of the Gazi University approved our study (24.12.18/961). Conventional Caldwell Luc operations requiring radical antrostomy, and other external approaches were not included in the study. Patients who had previously undergone surgery for IP were excluded from the study and only primary cases were included. The minimum follow-up period was 12 months. The patients who were diagnosed with malignancy ‒ Squamous Cell Carcinoma (SCC) – were excluded. In addition, Patients who had undergone both mucosal stripping and bone resection (curettage or drill) of tumor attachment site were included.

All patients had preoperative paranasal sinus tomography (PNCT). In addition to PNCT, patients were staged according to preoperative endoscopic examination and findings during surgery. The tumor involving sites in nasal cavity and paranasal sinuses and the attachment site of tumors were investigated from surgical data.

The Krouse classification was used as staging system (Table 1). There were no patients with T1 or T4 stage in the present study. T2 and T3 tumors were classified as early and advanced stage tumors, respectively. Moreover, we further analyzed impact of the stage and origin sites of tumors. Tumors attachments with the middle meatus and surroundings, middle turbinate and ostiomeatal complex were grouped as Lateral Nasal Wall (LNW) origin. Other origin sites of tumors were grouped as Medial Wall (MW) and Other Walls of Maxillary sinus (OWM).

All study subjects had undergone Endoscopic Sinus Surgery (ESS) as the main surgical procedure. Simple Tumor Resection (STR) was performed for early stage tumors that were limited in nasal cavity and did not exhibit Maxillary Sinus (MS) involvement. Additional endoscopic surgical procedures such as Extended Middle-Meatal Antrostomy (EMMA), endoscopic Caldwell-luc (ECL) and Endoscopic Medial Maxillectomy (EMM) had also been performed due to maxillary sinus involvement.

In the EMMA, the ostium of the maxillary sinus was extended into the posterior wall of maxillary sinus as the posterior border. The superior border was the insertion site of the Uncinate Process (UP), anterior border was the bone wall of lacrimal duct (lacrimal duct was protected) and the inferior border was the inferior turbinate (with resection of limited amount of inferior turbinate).

The endoscopic Caldwell-Luc operation, which involves a bone hole in the canine fossa large enough for endoscope and surgical instruments to reach to maxillary sinus, is distinguished from a formal Caldwell- Luc procedure that involves radical antrostomy. It was used as an auxiliary technique for EMMA in certain cases. Surgical instruments and endoscopes could complement each other with different combinations because of presence of two openings in different planes.

As the Endoscopic Medial Maxillectomy (EMM) procedure, a radical excision including the entire medial wall of maxillary sinus was performed. The limits of EMM were posterior wall of maxillary sinus as posterior border, the orbital floor as superior border, the floor of nose as inferior border and the anterior wall of maxillary sinus as anterior border. The angle of anterior and medial walls was excised to obtain adequate exposure. The mucosa of the maxillary sinus was then removed and the bone was drilled or curetted at attachment sites.

In all cases, the mass was completely removed along with mucosal and bone attachments. The surgical cavity was buffered for 2 days with antibiotic applied Merocel buffer.

### Statistical analysis

Statistical analyzes were performed with the IBM SPSS for Windows Version 19.0 package. Numerical variables were summarized with mean ± standard deviation. Categorical variables were shown by number and percentage. The normality of numerical variables was examined by the Kolmogorov–Smirnov test. *T*-test or Wilcoxon test was used for comparison of continuous variables depending on the normality of distribution. Differences among the categorical variables were evaluated by Chi-Square test. Differences among more than two groups were analyzed by one-way ANOVA when parametric test assumptions were obtained and by Kruskal Wallis test when parametric test assumptions were not available. Bonferroni correction was applied for post-hoc tests. Conventional clinical variables (age, gender etc.), tumor location and stage were entered in a multiple linear regression model with bootstrapping. The significance level was accepted as *p* < 0.05.

## Results

### Demographic datas and characteristic of tumors

In total, 55 (42 males ‒ 76.3 %) patients were included in the study. The mean age of the patients was 54.9 ± 14.4 years. According to the Krouse staging system, 37 patients were diagnosed as advanced stage (67.2 %) and 18 patients (32.7 %) as early stage (Table 1). The mean follow-up duration was 52.0 ± 39.4 months with a range of 12–144 months. The recurrence rate of the study was 21.8 % (12/55). The mean duration until first recurrence was 26.9 ± 17.5 months.

### Surgical outcomes according to Krouse classification system

All recurrences were observed in the advanced stages. Recurrence rate in advanced stage tumors was 32.4 %. There was a statistically significant relationship between tumor stages and recurrence rates. No recurrence was observed in patients who underwent STR or EMMA for early stage tumors. For advanced stage IP, EMMA, EMMA + ECL and EMM were performed and recurrence rates were 100 % (2/2), 53.8 % (7/13) and 13.6 % (3/22), respectively (Table 2).

**Table 2:** Recurrence rates and surgical distribution according to Krouse Classification System.

Stage	Surgical methods	Recurrence	Total recurrence
T2 (Early stage)	STR	0/10	0/18
	EMMA	0/8	
*p*-value		‒	
T3 (Advance stage)	EMMA	2/2^a^	12/37
	EMMA + ECL	7/13^a^	
	EMM	3/22	
*p*-value		0.005	
*p*-value			‒

STR, simple tumor resection; EMMA, extended middle meatal antrostomy; ECL, endoscopic Caldwell-Luc; EMM, endoscopic medial maxillectomy.

a *p *< 0.005.

### Surgical outcomes according to tumor origin sites and Krouse classification system

We analyzed our results based on originating sites of tumors by combining with Krouse staging system. The origin sites were classified as the LNW, MW and OWM. The number of tumors with LNW, MW and OWM origin were 10, 34 and 11, respectively. Recurrence rates for LNW, MW and OWM were 0 %, 29.4 % and 18.1 %, respectively (Table 3). In early stage LNW origination, no recurrence was observed in the STR group (0/10). In early stage MW origination, no recurrence was observed in EMMA group (0/8). In advanced stage MW origination, the recurrence rates of EMMA, EMMA + ECL and EMM were 100.0 %, 53.8 % and 13.6 %, respectively (*p* = 0.002). In advanced stage OWM origination, recurrence rates of EMMA+ECL and EMM were 20 % and 16.6 %, respectively (*p* = 0.887), (Table 3). Multivariate linear regression analysis revealed that the advanced stage tumor (*p* = 0.005) was the strongest independent variable associated with a recurrence.

**Table 3:** Recurrence rates and surgical distribution according to tumor origin site and Krouse Classification System.

Tumor origin site	Surgical methods	Krouse Classification System		Total recurrence
		Early stage	Advance stage	
LNW (except MW)	STR	0/10	‒	0/10
*p*-value		‒	‒	
MW of MS	EMMA	0/8	2/2^a^	10/34
	EMMA + ECL	‒	6/8^a^	
	EMM	‒	2/16	
*p*-value		‒	0.002	
OWM	EMMA + ECL	‒	1/5	2/11
	EMM	‒	1/6	
*p*-value		‒	0.887	
*p*-value				‒

LNW, lateral nasal wall; MW, medial wall; MS, maxillary sinus; OWM, other walls of maxillary sinus; STR, simple tumor resection; EMMA, extended middle meatal antrostomy; ECL, endoscopic caldwell-Luc; EMM, endoscopic medial maxillectomy.

a *p *< 0.005.

## Discussion

Sinonasal inverted papilloma is a benign epithelial tumor of the nasal cavity and paranasal sinuses. IP has a recurrence rate ranging from 10 % to 25.3 %.^6^ Surgery is the gold standard for treatment and complete surgical resection is the main aim to avoid recurrence.^11^ Recommended surgical method is to remove the whole mass and the attachment sites without any residue. In a study, it was found that mucosal excision of the origin site has not reduced the recurrence (52.2 %) however, excision of the bone tissue did (13 %).^12^ There are studies claiming that recurrence is primarily related with an incomplete resection of origin site rather than tumor nature. Recurrences were frequently observed in the tumor attachment sites indicated in previous surgeries.^13^, ^14^ Thus, the surgical techniques have changed from aggressive surgery to pedicle-oriented endoscopic surgery in the recent years.^15^ These data have led to questioning of existing staging systems that based on tumor volume.

Staging is an important factor for surgical outcomes. For this reason, several staging systems for IP have been described and commonly used over the last few decades.^16^, ^17^ These studies have been based on tumor volume and the collective notion of these studies was that large tumors have high recurrence risk. However, there are studies claiming that there is no association between tumor stage and recurrence rate according to different staging systems such as Krouse, Fruta and Citardi.^17^, ^18^, ^19^ Several studies have recommended staging systems considering origin site of tumor, thus origin based staging systems have been popular in recent years.^8^, ^20^ However, the classification that Krouse defined in 2000 is the most frequently used classification for IP.^17^ Recent studies have shown that the tumor stage or tumor origin site could be closely related to recurrence.^8^, ^9^, ^10^ We analyzed our surgical outcomes according to Krouse classification system and there was a statistically significantly difference between tumor stages in terms of recurrence. Nevertheless, the significant factor that affects recurrence was not only tumor origin site but also tumor volume and stage in the present study.

Although surgical techniques have been evolved to focus on a single tumor origination cite, maxillary sinus IP frequently have multifocal cite involvement.^21^ This is a warning that our surgical attention should not only focus on a single region. We should carefully examine not only the origination site but also other possible sites of tumor involvements. Therefore, classification systems that include both tumor origin site and tumor volume might be more useful. Our results showed that both tumor origin site and tumor stage were associated with recurrence. However, multivariate analysis revealed that tumor stage was the strongest predictor of recurrence.

Because it is hard to access all regions of the maxillary sinus by endoscopic approach alone, some studies have recommended combined endoscopic approaches as routine. Advanced endonasal approaches replaced external approaches such as the conventional Caldwell-Luc surgery. Endoscopic medial maxillectomy, endoscopic CL or endoscopic prelacrimal duct approaches could be used an auxiliary technique when ESS is insufficient for access to maxillary sinus lesion.^22^, ^23^, ^24^, ^25^, ^26^ In the present study, EMM and ECL were performed as auxiliary techniques to ESS.

ECL technique was used to remove attachment site of tumors that originates from MW and OWM by forceps or drill while the endoscopy were providing a view via EMMA.^26^ While this technique was successful for lateral, posterior and superior walls, it caused high recurrence in MW in the present study. The finding pointed out that EMM provided better vision than ECL for the inferior and anterior walls, and ensures removal of the tumor origin site for advanced stage MW tumors.^17^ According to our results, performing EMM for all OWM origin tumors may be an excessive surgery because EMMA + ECL was a successful method and less invasive technique for lateral, posterior and superior walls of MS. In a comprehensive study, which was based on a staging system depending on the origin site, authors suggested performing ESS alone for maxillary sinus IP covering posterior, lateral or superior wall origination.^8^ In the present study, no patient underwent ESS alone for the mentioned regions. Advanced technological instruments may be required for these regions to remove the attachment site of tumor by ESS alone. For MW attachment, Meng et al. recommended ESS-assisted prelacrimal duct approach in their staging system.^8^ Although authors suggest a specific surgery type for each tumor origination cite, our study showed that tumor stage and tumor volume have substantial importance on selecting the optimal surgery to avoid recurrence or excessive surgery. In our database, different type of surgeries such as EMMA, EMMA + ECL and EMM were performed for MW attachment site. EMMA was performed in tumors that MW attachment without OWM involvement (early stage according to Krouse) and showed low recurrence rates (0/8). Although EMMA or EMMA + ECL techniques were not alternative methods for the advanced stage MW origin tumors, performing EMM for all MW attachment site tumors might be an excessive surgery. Therefore, evaluating both the origin and volume of the tumor may be useful to prevent recurrence and excessive surgery.

### Limitations of study

Tumors originating from the sphenoid and frontal sinus were not included in this study since the number of patients included in the study was small. A more comprehensive prospective study with a longer follow-up that will include recurrence rates for each wall of maxillary sinus would provide better information about the topic.

## Conclusion

We analyzed the recurrence rates of endoscopic approaches for sinonasal IP that include 55 cases. Despite the fact that consideration of tumor origin site singly could lead to appropriate selection of the surgery type in most cases, tumor stage carries substantial importance in selection of surgery type for sinonasal-inverted papilloma. Therefore, an operation plan, which considers both tumor origin and tumor volume, may better aid surgeons in selecting optimal endoscopic surgical method rather than evaluating the tumor origin site singly. Selecting the endoscopic surgical surgery technique by this approach can prevent recurrence of IP or nonessential excessive surgeries.

## Funding

The authors received no financial support for the research, authorship, and/or publication of this article.

## Conflicts of interest

The authors declare no conflicts of interest.
